# Cost-Effectiveness of an Exercise Programme That Provided Group or Individual Training to Reduce the Fall Risk in Healthy Community-Dwelling People Aged 65–80: A Secondary Data Analysis

**DOI:** 10.3390/healthcare9060714

**Published:** 2021-06-10

**Authors:** Isaac Aranda-Reneo, Laura Albornos-Muñoz, Manuel Rich-Ruiz, María Ángeles Cidoncha-Moreno, Ángeles Pastor-López, Teresa Moreno-Casbas, Otago Project Working Group

**Affiliations:** 1 Economics and Finance Department, Faculty of Social Science, University of Castilla-La Mancha, 45600 Talavera de la Reina, Spain; Isaac.Aranda@uclm.es; 2 Nursing and Healthcare Research Unit (Investén-isciii), Instituto de Salud Carlos III, 28029 Madrid, Spain; lalbornos@isciii.es (L.A.-M.); MARIAANGELES.CIDONCHAMORENO@osakidetza.eus (M.Á.C.-M.); mmoreno@isciii.es (T.M.-C.); 3 Health Services Research on Chronic Patients Network (REDISSEC), Instituto de Salud Carlos III, 28029 Madrid, Spain; 4 Instituto Maimónides de Investigación Biomédica de Córdoba (IMIBIC), Universidad de Córdoba (UCO), Hospital Universitario Reina Sofía (HURS), 14004 Cordoba, Spain; apastor@uco.es; 5 CIBER on Frailty and Healthy Ageing (CIBERFES), Instituto de Salud Carlos III, 28029 Madrid, Spain; 6 Subdirection of Nursing, General Head Office of Osakidetza Bioaraba, 01006 Vitoria-Gasteiz, Spain

**Keywords:** cost-effectiveness, risk fall, older adults, randomized controlled trial, Otago Exercise Program, Tinetti, timed up and go, short physical performance battery, direct healthcare costs

## Abstract

Research has demonstrated that some exercise programs are effective for reducing fall rates in community-dwelling older people; however, the literature is limited in providing clear recommendations of individual or group training as a result of economic evaluation. The objective of this study was to assess the cost-effectiveness of the Otago Exercise Program (OEP) for reducing the fall risk in healthy, non-institutionalized older people. An economic evaluation of a multicenter, blinded, randomized, non-inferiority clinical trial was performed on 498 patients aged over 65 in primary care. Participants were randomly allocated to the treatment or control arms, and group or individual training. The program was delivered in primary healthcare settings and comprised five initial sessions, ongoing encouragement and support to exercise at home, and a reinforcement session after six months. Our hypothesis was that the patients who received the intervention would achieve better health outcomes and therefore need lower healthcare resources during the follow-up, thus, lower healthcare costs. The primary outcome was the incremental cost-effectiveness ratio, which used the timed up and go test results as an effective measure for preventing falls. The secondary outcomes included differently validated tools that assessed the fall risk. The cost per patient was USD 51.28 lower for the group than the individual sessions in the control group, and the fall risk was 10% lower when exercises had a group delivery. The OEP program delivered in a group manner was superior to the individual method. We observed slight differences in the incremental cost estimations when using different tools to assess the risk of fall, but all of them indicated the dominance of the intervention group. The OEP group sessions were more cost-effective than the individual sessions, and the fall risk was 10% lower.

## 1. Introduction

Falls are one of the main causes of hospitalization among elderly people [1,2,3]. Most fall-related injuries are minor, such as bruises, lacerations, strains, and sprains, but some can have serious long-term consequences. They can even cause more serious injuries, such as joint dislocations, fractures, and concussion [4]. About 10% of falls result in a fracture, and these have been identified as a major source of morbidity and mortality in older people [5].

In 2014, the Centers for Disease Control and Prevention estimated that every year 27,000 people aged 65 plus die from falls in the USA and that they also lead to 2.8 million annual emergency room visits and 800,000 hospital stays [1]. A 2015 population-based study by Florence et al. (2018) estimated that the cost of treating falls in the same age group in the USA was around USD 49.5 billion a year [6]. Papers published in 2010 and 2015 reported that falls cost the European Union EUR 25 billion in direct healthcare costs per year [7,8,9].

Most falls could be prevented, including those in inpatient settings [10]. Most of the interventions that aim to prevent falls can be classified according to the taxonomy developed by the European Network for the Prevention of Falls (ProFANE). Interventions are often based on known and modifiable risk factors for falls and deficits in gait and balance. This is why some of the fundamental elements that can be found in fall prevention programs include gait training, balance, and other strength and resistance exercises, flexibility exercises, and three-dimensional training, such as Tai Chi [11,12].

Research to date has demonstrated that some exercise programs are effective in reducing fall rates in community-dwelling older people. These were mainly exercises that challenged their balance and provided a higher total quantity of at least three hours of exercise per week [13,14,15,16,17,18,19,20]. These programs produced positive effects on both mobility and physical functioning.

In addition, several studies have demonstrated that preventive group sessions can significantly reduce the rate and risk of falling [14]. Moreover, several authors have highlighted that both individual and group exercise programs are equally effective for older people aged 65–80 years [14,18,21,22,23,24]. However, studies carried out on older people who have a higher risk of falling have showed conflicting results on whether group training is more effective than individual training [25,26]. Gillespie LD et al. (2012) reported in their systematic review that individual and group exercise in different categories could reduce falls or risk of falling [14]; however, most studies have not integrated different modalities in the same trial using a non-inferiority methodology and have not evaluated economic results.

Regarding the economic aspects, group sessions are also better value for money, as one instructor can train a number of older people at the same time. However, cost-effectiveness analysis studies of these group exercise sessions have focused on the economic evaluation of these sessions versus usual care, rather than comparing individual versus group exercise programs [27]. That is why our aim was to assess whether group exercise programs, not only reduce healthcare costs, but also improve the health of older adults by reducing their risk of falling.

## 2. Materials and Methods

### 2.1. Study Design

**Figure 1:**
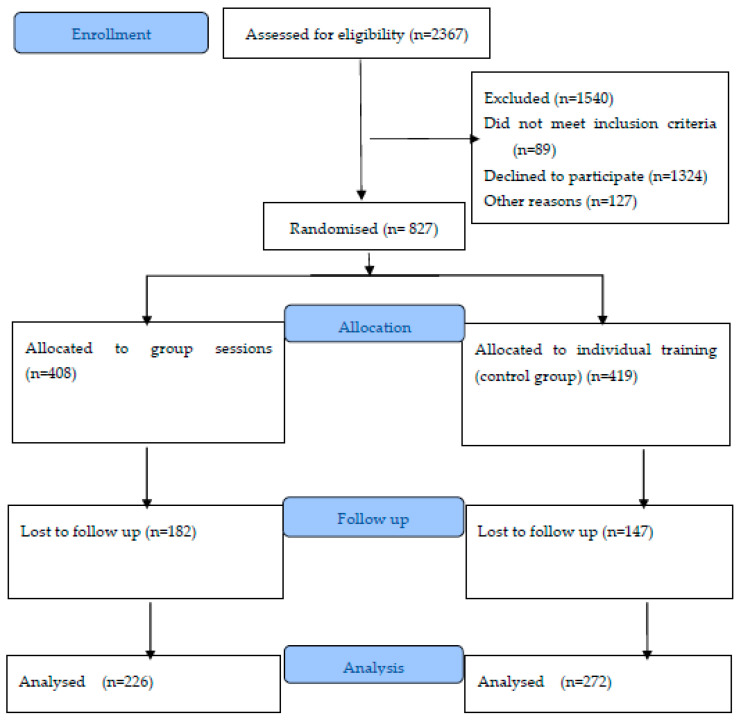
Consort Flow Diagram (2010).

This study was a secondary data analysis of data collected in a non-inferiority multi-center intervention controlled clinical trial in a community-dwelling 65- to 80-year-old population (Figure 1). The study was recorded at ClinicalTrials.org (NCT03320668). More details about the trial design have previously been published [28]. Briefly, the participants aged 65–80 were consecutively enrolled from 21 Spanish primary healthcare centers between January 2017 and December 2019. The centers were based in eight regions across Spain. All the participants were still living in the community, rather than in institutions, could walk independently, and provided signed, informed consent.

We excluded people who had been living outside the area covered by the primary health center for more than 9 months or who had a life expectancy of less than 9 months. The exclusion criteria also included participants with moderate or severe cognitive impairment and/or sight or hearing impairments that would prevent them from following the group sessions. Those with absolute contraindications for performing physical exercise were also excluded. We also excluded people who were already participating in another clinical trial, research study, or exercise program where they performed balance and strength activities that were similar to the group sessions. All the participants continued to follow the advice provide by health professionals to control their health, including any treatments.

### 2.2. Group and Individual Interventions

All participants received the Otago Exercise Program (OEP), [29] which was developed to reduce falls in community-dwelling people aged 65 plus. It comprises 24 strength and balance exercises, with ongoing support to help them to continue the program at home. The group training consisted of 5 sessions, 4 over eight weeks, with a reinforcement session at six months. The OEP was taught by qualified OEP later life training instructors, as previously described [28]. The individual and group training sessions were identical, apart from the number of people taking part. They were both provided at the participants’ local primary care health center and they followed the same recommendations at home, according to the protocol. The group arm comprised 6–12 participants, and the healthcare professionals leading the sessions were supported by a colleague. Both had undergone the OEP later life training program. The group sessions were only considered complete if the subject had completed all of the 5 sessions, and those that did not were recorded as losses in the analysis. Due to potential losses in relation to the previous criteria, in addition, an intention to treat analysis was carried to compare the results of both approaches (protocol and intention to treat analysis).

### 2.3. Variable Collection

We used questionnaires to collect information from the participants at baseline and after 12 months. These comprised the timed up and go test (TUG), the modified Tinetti scale, and the short physical performance battery (SPPB) test. The participants also completed the exercise adherence rating scale [28,30]. We also extracted details on the primary and specialized care resources the participants required during the 12-months to follow up from their clinical record. In addition, the participants recorded in an exercise log for confirmation of completing the intervention.

### 2.4. Economic Assessment

We carried out the economic evaluation from a healthcare provider perspective, by comparing the costs and effects from baseline to the 12-month visit. This meant that only direct healthcare costs, together with the group sessions costs, were included in the analysis. The primary outcome measure was the risk of falling, which was assessed using the TUG results at the 12-month visit [31,32]. TUG was validated in community dwelling older adults population [33]. A secondary analysis was carried out using the recommended thresholds for the Tinetti [34] and SPPB test [35] scores, which indicated the fall risk. We inverted the scales so that positive health effects could be related to cost-effectiveness. This means that instead of informing the risk of fall, we obtained the non-risk of falling. Therefore, the health effect obtained with the intervention was considered as a positive health effect, and the intervention was value for money when gaining a lower risk of falling and needing lower healthcare resources. This analysis is characterized by the fact that the health outcomes are expressed in the form of units commonly used in clinical practice, which are very common and easier to interpret. We wanted to know whether the group sessions were less costly and more effective than providing individual training sessions. Therefore, the positive health result of the group training sessions was to increase the number of people not at risk of falling.

The direct healthcare costs were based on unit costs from official regional sources. This enabled us to estimate the daily costs of hospitalization, visits to family doctors, and visits to hospital emergency departments or primary care health centers. We applied a micro-costing approach, which means that we multiplied the unit costs by the healthcare resources collected from the questionnaires. The costs included the OEP later life training fee [36] for the health professionals involved in all the sessions and all the resources needed to deliver them. Moreover, we assessed the time employed by healthcare professionals based on the number of healthcare professionals involved and the duration of the sessions. In this sense, we used the wages of healthcare professionals obtained from the site where the participants were enrolled to value the time provided by healthcare professionals to deliver the intervention. The recruitment sites recorded the individual and group session durations. We used these records to assess the real duration of sessions in both arms in the economic assessment. All costs were expressed in Euros using 2019 prices. As the follow-up period was only 1 year, discounting costs and effects were not necessary.

### 2.5. Statistical Analysis

Incremental cost-effectiveness ratios (ICER) were calculated by dividing the adjusted mean direct healthcare cost differences by the health effects (see Formula 1). The ICER reveals the incremental cost per health effect gained (reducing the risk of fall) of switching from the control group to the intervention group. In other words, how much the national health service should spend to gain this health effect. The main analysis used the timed up and go test results obtained during the 12-month visit to estimate the health effects. The secondary analysis considered the risk of fall as a health effect, by using the Tinetti and SPPB test results. The cost-effectiveness analysis was adjusted using a seemingly unrelated regression model. This approach allowed us to adjust the costs and health effect uncertainty due to unobserved factors that could affect the costs and health effects [37,38,39]. This meant that the differences in costs and health effects were adjusted for sex, age, the level of education the participants’ had reached, the recruitment site, and the Tinetti values at baseline. The cost and effect seemingly unrelated regression difference estimates between the group sessions and individual control sessions were bootstrapped to 5000 replications. Then, they were plotted on cost-effectiveness planes to treat the uncertainty regarding the health and costs estimations. Cost-effectiveness acceptability curves were also presented to show the probability that the group sessions would be considered as cost-effective at different thresholds. These were based on society’s willingness to pay for a gain in the health effect for each outcome considered [40].

(1) ICER=Costintervention−Cost controlHealth effectsinterventionHealth effectscontrol

Formula (1): Incremental cost-effectiveness ratio (ICER).

## 3. Results

We recruited 2367 participants and 827 met the inclusion criteria and agreed to participate. Of these, 498 participants completed the follow up at 12 months: 226 in the group sessions and 272 in the individual control sessions (Figure 1). They had a mean age of 71.9 ± 4.1 years old at baseline, 68% were female and 64% were married. Most (45%) of the participants had finished primary studies, while only 41 (8%) had university degrees. There were no remarkable differences between the study groups at baseline, except for sex. Table 1 shows that there was a high percentage of female participants in the group sessions, but this difference was not statistically significant (χ2 = 3.26; *p* = 0.071). We also noted that there was higher mobility in the SPPB test scores in the group sessions, but this difference was not statistically significant (t(496) = −1.54; *p* = 0.125).

To assess the effect of subjects who did not complete the study, we analyzed the subjects who fell and who had falls records available (805 at baseline). We found no difference in falls between the groups (*p* > 0.05), as well as in protocol analysis (*p* > 0.05).

The cost per patient was clearly lower (−€51.28; 95% CI: −54.81 to −47.75) when participants took part in the group sessions rather than the individual control sessions. During the 12-month follow-up period, there were no statistically significant differences in the healthcare costs between both groups. The patients who took part in the group sessions had higher costs for visits to their family doctor, but lower costs for hospital visits (Table 2). When we used the seemingly unrelated regression approach, this showed that the cost reductions per patient for the group, rather than the individual sessions, were statistically significant at the 12-month follow-up visit (−€52.35; 95% CI: −62.49 to −42.22) (Table 3).

When it came to the health effects of the sessions, the differences between the group and individual sessions were only statistically significant in the adjusted difference analysis for the timed up and go and SPPB tests (Table 3).

**Figure 2:**
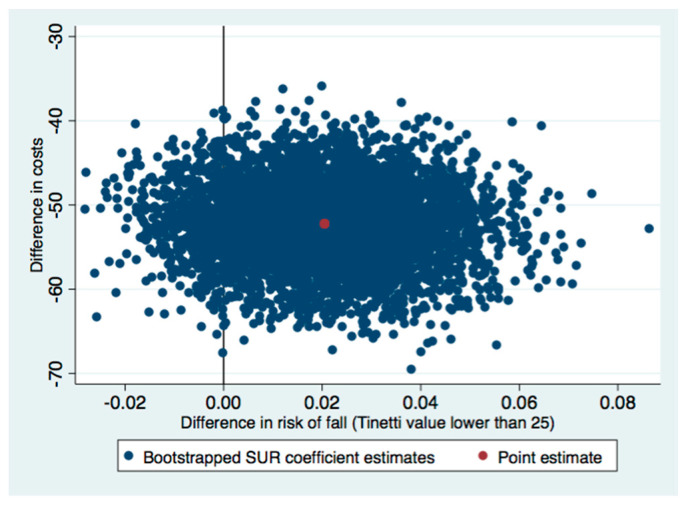
Cost-effectiveness plane for risk of fall using the Tinetti instrument. SUR: seemingly unrelated regression.

**Figure 3:**
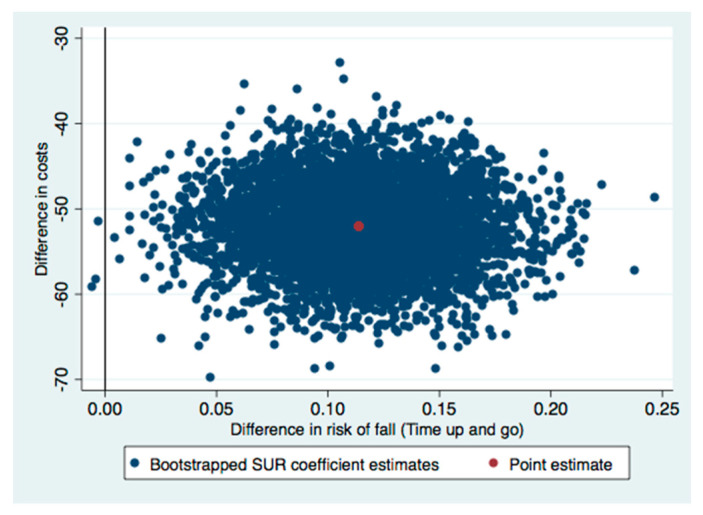
Cost-effectiveness plane for risk of fall using the timed up and go instrument. SUR: seemingly unrelated regression.

**Figure 4:**
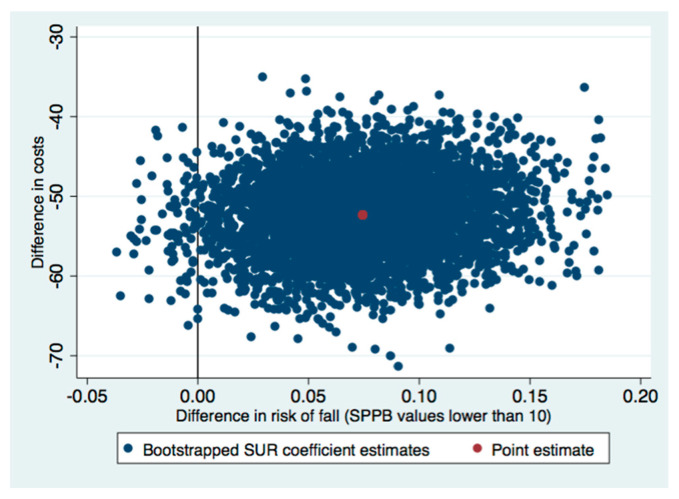
Cost-effectiveness plane for risk of fall using the SPPB Test. SUR: seemingly unrelated regression.

**Figure 5:**
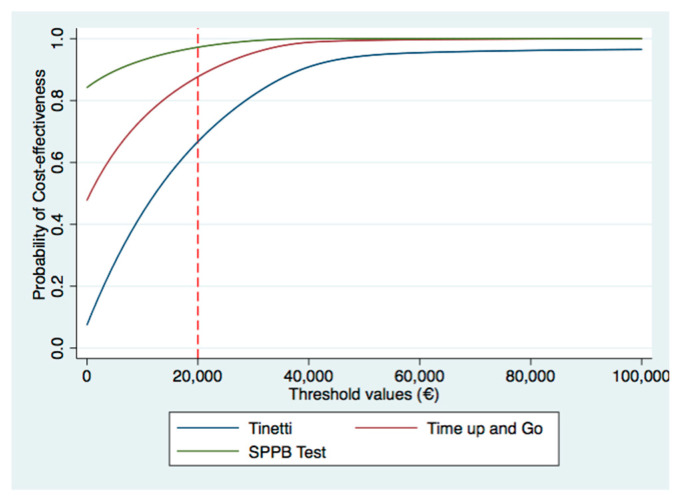
Cost-effectiveness analysis curves for the different outcomes.

The ICERs indicated that the group sessions were better than the individual control sessions. This means that switching from the control to the intervention involve cost-savings. Therefore, national health services could expect that delivering the OTAGO program in group sessions would give lower costs and a lower risk of falling than individual sessions. Regarding the uncertainty of the health gains and cost estimations, most of the incremental cost-effectiveness pairs were located in the southeast quadrant of the cost-effectiveness plane (see Figure 2, Figure 3 and Figure 4). Most of the replications resulting from the SUR models fell in the southeast quadrant, which means fewer costs and more health effects gained. However, there was more uncertainty if we used the SPPB or Tinetti scales (Figure 2 and Figure 4) to estimate whether there was no risk of falling, since some replications fell in the southwest quadrant (less costly but a lower health effect gained). In line with this uncertainty observed in the cost-effectiveness plane, the CEACs analysis showed that the probability of being cost-effective on a willing-to-pay of €20,000 differed by the assessment of the risk of fall. It was 100% when we used the SPPB test, 81% with the Tinetti assessment, and 97% when the timed up and go test was used (Figure 5). Thus, although the intervention surpassed the control independently of the outcome, we chose to assess the health gains, as considering the effectiveness of the intervention with the Tinetti scale seemed to be more uncertain.

## 4. Discussion

Our study, which used a range of tools (Tinetti, TUG, and SPPB), indicated that the group sessions were more cost-effective and delivered greater health benefits than the individual sessions. This was because they reduced the risk of falls and the healthcare costs associated with falls. Therefore, from a healthcare system perspective the group sessions can improve health compared to the individual sessions, and the health benefits gained can be achieved with lower resources (leading to lower costs).

When it came to the health effects of the sessions, the risk of falls in the intervention group was lower than the control group. Regarding the cost per patient, this was also clearly lower in the group sessions rather than the individual. In addition, this seems to be the main reason that explains the cost savings in the intervention group. However, the cost reductions per patient for the group sessions, rather than the individual, were also lower at the 12-month follow-up visit. Finally, it should be noted that all incremental cost-effectiveness ratios indicated that group sessions were dominant, meaning that group sessions were more effective and less costly than individual sessions and that adopting this approach could lower healthcare costs in clinical practice.

However, these savings were lower than those reported by a number of previous studies. For example, Albert et al. (2016) reported that using the Healthy Steps for Older Adults (HSOA) fall prevention program reduced average hospitalization costs by USD 840 per person [41]. In addition, Keall et al. (2017) reported a 33% (95% CI 5% to 49%) reduction in home fall injury costs when the Home Safety Assessment and Modification program was used [42]. However, it should be highlighted that the nature of these interventions was also very different. In addition, we can explain the differences between these studies and ours by looking at the economic evaluation methods used by the other authors and the healthcare resources included in the analyses. Keall et al. (2017) included all subsequent visits related to the fall, together with the social costs due to lost working days, in their analysis. In contrast, we could only include direct healthcare costs and our participants were not working. On the other hand, the model by Albert et al. (2016) included all the emergency department and hospitalization costs during the follow-up period, instead of just those related to the fall. This program proved to be cost-effective only for more healthy patients.

Regarding the healthcare costs during the follow-up, they were very similar in both groups, and the group sessions did not achieve notable savings during this period, with regard to factors such as hospital admissions and visits. The absence of significant savings in our study was consistent with those of the ProAct65+ study, [43] a pragmatic, three-arm parallel design that evaluated the OEP, the falls management exercise program (FAME), and standard care. When the researchers compared the OEP with standard care, they did not find any statistically significant differences in most of the costs, per participant, of the primary care services related to family doctors or practice nurses. However, the total cost of usual care was higher than the OEP. Most of the OEP interventions discussed and compared with our study were based on original research, including specific training sessions and home-based exercise [29].

Our intervention costs were found to be lower than those reported in previous studies. Abdulrazaq et al. (2018) estimated that providing the OEP to community dwelling adults aged 18 plus with rheumatoid arthritis cost GBP 217.72 (USD 314.34) per person derived from the application of OEP in community dwelling adults with rheumatoid arthritis from ≥18 years [44]. The FAME was more expensive than the OEP when it was delivered with peer mentors, as the respective costs ranged from GBP 269–218 versus GBP 88–117 per participant [43]. We found even higher costs for a similar program that provided strength and balance exercises. The reported cost of the SUNBEAM program was USD 463 per participant [45]. However, these differences may have been due to the different contexts in which the studies were conducted, and the unit costs used to assess the cost of delivering the programs.

In terms of healthcare costs, implementing the OEP delivered more than a 100% return of investment. This means that for every GBP 1 (USD 1.44) spent on healthcare GBP 1.01 (USD 1.46) would be returned. The move with balance program for institutionalized older people reported a similar return on investment of 1.7:1 [46]. Another study that measured the effectiveness of the OEP showed a 35% reduction in the number of falls and fall-related injuries [47].

The ICERs in our study indicated that the group sessions dominated the individual control sessions and that the most incremental cost-effectiveness pairs were located in the southeast quadrant of the cost-effectiveness plane. This means that the group sessions were more effective and less costly than the individual control sessions. Numerous studies have quantified the ICER of programs in terms of cost per quality-adjusted life year (QALY) [47,48,49,50,51,52,53]. However, we could not include a health-related quality of life (HRQOL) estimate in our study. Nevertheless, the lower risk observed in the people who received the group sessions might lead us to expect that this group would also have a higher health-related quality of life. Other authors have reported increments in HRQOL when participants with diabetes and frailty achieved higher results in the SPPB test when they participated in a multimodal intervention that focused on physical exercise [54]. Therefore, we could expect similar effects in our study participants.

Regarding the probability of being cost-effective on a willing-to-pay (WTP) basis, previous studies note that the cost-effectiveness in WTP of an intervention depended on age [55]. The interventions in that study were cost-effective at a higher willingness to pay levels (≥USD 25,000) for adults aged 75–84 years and at a lower willingness to pay (<USD 5000) for adults aged 85+ years. Our analysis was adjusted for age and sex because previous studies suggested that there were differences in balance performance between genders and some of these were only significant in elderly people. They also suggested that these gender differences in age-related changes in balance performance were demonstrated in a mediolateral direction. These gender differences may also contribute to the gender differences in balance-related problems, such as falls [56]. It has also been reported that men had a lower level of postural stability than women [57]. Another study highlighted the presence of age and gender differences in objective measures of physical capability and also demonstrated that it is possible to harmonize data from several large cohort studies [58].

Our results indicate that the study objective was met and suggest the incorporation of exercise programs in the elderly population to reduce health care costs in clinical practice. However, some limitations should be pointed out. The economic evaluation only considered the healthcare costs from a national health service or healthcare provider perspective in the cost analysis, because differences could have appeared in the recommendations for funding the technology assessed [59,60]. It was not possible to include the non-healthcare costs, such as any informal care the person required and the costs of professionals, such as occupational therapists and physiotherapists. However, we do not think that these extra costs would have affect the results, due to the strong costing differences. On the other hand, other studies recommended measuring health-related quality of life as a health effect to be assessed [61,62]. We felt unable to include a validated tool. However, as mentioned above, we anticipated that the health-related quality of life of the people who participated in our group sessions would have improved, as other studies that reported high SPPB results also reported quality of life improvements [63,64]. In relation to the risk of falling measure, we did not include dietary behaviors; however, the body mass index variable was taken into consideration. Another limitation considered was the potential variability within centers and the professionals who led the interventions, however a high effort at coordination was made, and a cascade training model accredited by Later Life Training was provided. Regarding the sample size used in this study, it was calculated using the risk of fall as the main outcome instead of the potential cost savings generated with the intervention or the ICER results. Other authors have discussed the relevance of using economic-related outcomes to estimate the power size, doubting that this approach was useful [65,66,67]. Finally, although we did not achieve our target sample size due to losses, this study included a large sample of an exercise intervention trial and we performed protocols and intention to treat analyses and did not find any differences.

## 5. Conclusions

The cost savings generated by providing OEP group sessions, rather than individual sessions, in healthy community-dwelling people aged 65–80, were clear. Most of the savings were due to lower session costs, but there were also cost savings due to reduced hospitalizations. In addition, the people who took part in the group sessions had a 10% lower fall risk, according to the timed up and go test. These findings support the benefits of providing groups sessions from a national health services perspective. As far as we know, there has been no previous cost-effectiveness analysis of the OEP intervention that has revealed how much healthcare providers could gain delivering this program in group sessions. Decision-makers (public and private) could use these novel findings to drive reforms in caring for older people aged 65–80 years from a more proactive perspective that empowers patients and makes them feel one of the stakeholders involved in the health production function of health systems. However, further research that considers a broader costs analysis perspective (a societal perspective that includes informal care costs) and incorporating health-related quality of life outcomes is required, to provide a complete description of the potential benefits of recommending delivering the OEP in groups.

## Figures and Tables

**Table 1:** Participants’ characteristics at baseline.

	Control (n = 272)	Intervention (n = 226)
Age, mean (SD)	71.67 (4.11 *)	72.17 (4.18 *)
Modified Tinetti Scale, mean (SD)	32.06 (3.94 *)	31.68 (4.24 *)
SPPB Test, mean (SD)	9.9 (1.9 *)	10.17 (1.97 *)
**Sex, n (%)**		
Male	99 (36)	65 (29)
Female	173 (64)	161 (71)
**Education level, n (%)**		
None	26 (10)	17 (8)
Did not finish primary studies	52 (19)	54 (24)
Primary studies (0–11/12 years)	122 (45)	104 (46)
Secondary studies (11/12–17/18 years)	43 (16)	39 (17)
University studies	29 (11)	12 (5)
**Marital status, n (%)**		
Single	17 (6)	11 (5)
Married	177 (65)	141 (62)
Widow/widower	65 (24)	63 (28)
Other	13 (5)	11 (5)

* SD = standard deviation.

**Table 2:** Costs and outcome differences of the group and individual sessions at the 12-month visit (unadjusted).

**Costs in Euros**	**Intervention Mean (SD) (€)**	**Control Mean (SD) (€)**	**Mean Difference (95% CI) ^a^(€)**
Visits to family doctors	5.74 (21.24)	4.11 (17.78)	1.63 (−1.8; 5.07)
Hospital costs	7.82 (42.84)	8.46 (54.94)	−0.64 (−9.45; 8.17)
Direct healthcare costs	13.56 (54.1)	12.57 (58.36)	0.99 (−9; 10.98)
Intervention costs	24.47 (10.24)	75.75 (25.36)	−51.28 (−54.81; −47.75)
Total costs	38.03 (55.85)	88.32 (62.17)	−50.29 (−60.79; −39.79)
**No-risk of Falling**	**Intervention Mean (SD)**	**Control Mean (SD)**	**Mean Difference (95% CI) ^b^**
Modified Tinetti	0.98 (0.15)	0.96 (0.19)	0.01 (−0.02; 0.04)
Timed Up and Go	0.80 (0.43)	0.71 (0.46)	0.09 (0.01; 0.17)
SPPB	0.79 (0.41)	0.75 (0.44)	0.04 (−0.03; 0.12)

(€) Euros; ^a^ negative value indicates lower costs in the group sessions arm; ^b^ positive value indicates lower risk of falls in the group session arm. SD = standard deviation.

**Table 3:** Incremental costs and health effects during the 12-month follow up adjusted for age, sex, education level, study site, SPPB, and Tinetti results at baseline (*p*-value < 0.05).

Outcome	Incremental Costs in Euros (€) ^a^	Incremental Effects ^a^	ICER
Tinetti ^a^	−52.19 (−61.46 to −42.92)	0.02 (−0.01 to 0.05)	Dominant
Timed Up and Go	−52.19 (−61.50 to −42.88)	0.11 (0.05 to 0.18)	Dominant
SPPB ^b^	−52.35 (−62.32 to −42.06)	0.08 (0.01 to 0.14)	Dominant

(€) Euros; ^a^ Tinetti values higher than 25 indicated no fall risk. ^b^ SPPB scores higher than 10 indicated no fall risk. ICER = incremental cost-effectiveness ratio.
